# Associations Among School Absenteeism, Gastrointestinal and Respiratory Illness, and Income — United States, 2010–2016

**DOI:** 10.15585/mmwr.mm6853a1

**Published:** 2020-01-17

**Authors:** David Berendes, Ashley Andujar, Lisa C. Barrios, Vincent Hill

**Affiliations:** ^1^Division of Foodborne, Waterborne, and Environmental Diseases, CDC; ^2^Division of Adolescent and School Health, CDC.

Control of communicable diseases in children, including respiratory and diarrheal illnesses that affect U.S. school-aged children, might require public health preventive efforts both in the home and at school, a primary setting for transmission. National Health Interview Survey (NHIS) data on school absenteeism and gastrointestinal and respiratory illnesses in the United States during 2010–2016 were analyzed to examine their associations with income. Prevalence of gastrointestinal and respiratory illnesses (queried for the 2 weeks preceding the survey) increased as income decreased. The likelihood of missing any school days during the past year decreased with reduced income. However, among children who missed school, those from low-income households missed more days of school than did children from higher income households. Although the reason for absenteeism cannot be ascertained from this analysis, these data underscore the importance of preventive measures (e.g. hand hygiene promotion and education) and the opportunity for both homes and schools to serve as important points for implementation of public health preventive measures, including improved hand hygiene practices.

Data from the 2010–2016 NHIS (*1*) were obtained from IPUMS Health Surveys (https://nhis.ipums.org/nhis) and analyzed. NHIS is an annual, national household survey on the health of the civilian noninstitutionalized U.S. population, administered continually throughout the year. Family income data were linked to information about the school-aged child (5–17 years) with regard to 1) any school absenteeism in the last year, 2) number of days (reported as an integer) absent in the last year, and 3) gastrointestinal illness or respiratory illness (occurrence of a cold) during the 2 weeks preceding the interview. NHIS-imputed income files were used to present estimates by income brackets and by whether the family lived at or above the federal poverty level.* The statistical software R (version 3.4.3, R Foundation for Statistical Computing) was used account for the complex sample design and weighting of the survey and compare school absenteeism, illness, and income using linear and logistic regression models, unadjusted and adjusted for age and sex of the child and year of survey. P-values <0.05 were considered statistically significant.

A total of 61,482 (9.6%) households selected for the NHIS were sampled for the child-specific questionnaire and provided data about their school-age child’s health and days of school missed. Households varied across income categories, with 30% earning <$35,000 per year and 21% below the federal poverty level (Table 1). Sixty-nine percent of children missed ≥1 day of school in the previous year, and approximately 15% missed ≥6 days (mean = 3.24 days per child). The prevalence of gastrointestinal and respiratory illness in the past 2 weeks was 5% and 13%, respectively.

**TABLE 1:** Reported income, school absence, and gastrointestinal and respiratory illnesses among children aged 5–17 years — United States, 2010–2016*

Characteristic	No. of respondents (%)							
**Year**							
**2010**	**2011**	**2012**	**2013**	**2014**	**2015**	**2016**	**Total**
**Below FPL^†^**	1,638 (20.6)	1,898 (20.6)	2,022 (20.8)	1,923 (21.2)	1,986 (21.8)	1,680 (19.8)	1,230 (18.7)	**12,386 (20.5)**
**Annual income**								
<$34,999	2,757 (31.6)	3,132 (31.5)	3,296 (31.1)	3,091 (30.5)	3,112 (30.4)	2,674 (28.3)	2,041 (27.1)	**20,103 (30.1)**
$35,000–$49,999	1,092 (12.8)	1,296 (13.8)	1,240 (13.1)	1,264 (12.5)	1,277 (12.5)	1,149(12.7)	927 (11.8)	**8,245 (12.7)**
$50,000–$74,999	1,382 (17.9)	1,503 (17.1)	1,568 (16.7)	1,512 (17.1)	1,587 (16.5)	1,529 (16.8)	1,330 (16.2)	**10,411 (16.9)**
$75,000–$99,999	937 (13.0)	1,047 (12.6)	1,192 (13.5)	1,117 (13.1)	1,226 (12.6)	1,058 (12.6)	1,093 (13.1)	**7,670 (12.9)**
≥$100,000	1,701 (24.7)	1,942 (25.1)	2,056 (25.6)	2,141 (26.7)	2,348 (28.0)	2,323 (29.6)	2,543 (31.9)	**15,054 (27.4)**
**School days absent during previous year^§^**								
0	2,275 (27.0)	2,722 (28.8)	3,230 (34.0)	2,849 (29.9)	3,099 (32.1)	2,700 (29.9)	2,410 (32.7)	**19,285 (30.6)**
Any	5,594 (73.0)	6,197 (71.2)	6,122 (66.0)	6,275 (70.1)	6,452 (67.9)	6,033 (70.1)	5,524 (67.3)	**42,197 (69.4)**
1–2	2,150 (28.0)	2,524 (29.8)	2,725 (29.5)	2,627 (29.9)	2,779 (29.7)	2,553 (30.4)	2,364 (30.1)	**17,722 (29.6)**
3–5	2,136 (27.7)	2,365 (26.6)	2,207 (24.0)	2,353 (26.3)	2,421 (26.0)	2,157 (25.2)	2,005 (23.7)	**15,644 (25.6)**
6–10	857 (11.4)	874 (10.1)	811 (8.7)	866 (9.5)	866 (8.6)	900 (10.3)	788 (9.3)	**5,962 (9.7)**
≥11	451 (5.9)	434 (4.6)	379 (3.8)	429 (4.5)	386 (3.6)	423 (4.2)	367 (4.3)	**2,869 (4.4)**
Mean days absent (SD)	3.73 (7.61)	3.35 (6.65)	2.96 (6.54)	3.27 (6.18)	3.00 (6.33)	3.24 (6.09)	3.10 (6.05)	**3.24 (6.52)**
**Illness during past 2 weeks**								
Gastrointestinal	413 (5.8)	470 (5.6)	399 (4.5)	437 (5.1)	476 (5.0)	392 (4.0)	371 (4.6)	**2,958 (5.0)**
Respiratory	1,041 (13.1)	1,255 (14.5)	995 (10.8)	1,299 (14.1)	1,210 (12.9)	1,111 (12.2)	997 (12.2)	**7,908 (12.8)**

**Abbreviations:** SD = standard deviation; FPL = federal poverty level.

* Data are presented as the unweighted number of respondents (N) and weighted percentage (%), with the exception of the mean number of school days absent (weighted mean and standard deviation presented).

^†^ FPL represents an indicator used to define the boundary for those eligible for federal aid; FPL is defined by the U.S. Department of Health and Human Services annually each January to adjust for inflation and is proportional to the size of the household.

^§^ Parents or caregivers reported the number of days missed as integers, which were then categorized into the categories shown.

Reported school absence during the previous school year and reported respiratory or gastrointestinal illness during the previous 2 weeks were categorized by family income (Table 2). Compared with children in each of the other income categories, children in the lowest income bracket households (earning <$35,000 per year) had a lower likelihood of missing school during the previous year (65% versus 67%–73%) and higher prevalence of gastrointestinal illness (6% versus 4%–5%) and respiratory illness (14% versus 12%–13%) in the previous 2 weeks. Adjusting for age, sex, and year of survey, children in the lowest income bracket were 3%–12% (95% confidence intervals [CIs] ranged from 1%–14%) less likely to miss school, but 18%–31% (95% CIs ranged from 2%–39%) more likely to have had a recent gastrointestinal illness. Children in the lowest income bracket were also 6%–15% more likely to have had a respiratory illness, although comparisons with the $50,000–$74,999 and >$100,000 income brackets were not statistically different.

**TABLE 2:** Reported absence and illness by income and poverty status among children aged 5–17 years and percentage of respondents reporting school absence and illness among children aged 5–17 years, by income and federal poverty level (FPL) status* — National Health Interview Survey, 2010–2016, United States, 2010–2016†

Characteristic	No. of respondents (%)						
	Income					Poverty status	
	<$35,000	$35,000–$49,999	$50,000–$74,999	$75,000–$99,999	≥$100,000	Below FPL	At or above FPL
**School days absent**							
0	7,105 (34.6)	2,741 (32.6)	3,180 (30.4)	2,154 (27.8)	4,105 (26.8)	4,423 (34.9)	14,862 (29.5)
Any	12,998 (65.4)	5,504 (67.4)	7,230 (69.6)	5,516 (72.2)	10,949 (73.2)	7,953 (65.1)	34,244 (70.5)
PR (95% CI)	Referent	1.03 (1.01 to 1.05)	1.06 (1.04 to 1.09)	1.10 (1.08 to 1.13)	1.12 (1.10 to 1.14)	Referent	1.08 (1.06 to 1.10)
aPR^§^ (95% CI)	Referent	1.03 (1.01 to 1.06)	1.07 (1.04 to 1.09)	1.11 (1.08 to 1.13)	1.12 (1.10 to 1.14)	Referent	1.09 (1.07 to 1.11)
1–2	4,774 (24.0)	2,231 (28.2)	3,052 (29.7)	2,408 (31.8)	5,257 (35.4)	2,864 (23.6)	14,858 (31.2)
3–5	4,779 (24.3)	2,049 (24.5)	2,715 (26.2)	2,122 (27.6)	3,979 (26.4)	2,943 (24.4)	12,701 (26.0)
6–10	2,154 (10.7)	796 (9.8)	1,043 (10.0)	723 (9.6)	1,246 (8.4)	1,328 (10.5)	4,634 (9.5)
≥11	1,290 (6.4)	428 (4.9)	420 (3.8)	264 (3.2)	467 (3.0)	818 (6.5)	2,051 (3.9)
Mean (SD) all	3.73 (8.27)	3.29 (6.60)	3.05 (5.36)	2.95 (4.43)	2.91 (5.68)	3.76 (8.61)	3.10 (5.85)
Est^¶^ (95% CI)	Referent	−0.44 (−0.67 to −0.21)	−0.69 (−0.89 to −0.48)	−0.78 (−0.99 to −0.57)	−0.82 (−1.03 to −0.62)	Referent	−0.66 (−0.88 to −0.44)
aEst^§^ (95% CI)	Referent	−0.46 (−0.69 to −0.22)	−0.71 (−0.91 to −0.50)	−0.80 (−1.00 to −0.59)	−0.84 (−1.05 to −0.64)	Referent	−0.69 (−0.91 to −0.47)
Mean (SD)**	5.70 (9.66)	4.88 (7.54)	4.37 (5.96)	4.09 (4.75)	3.98 (6.31)	5.78 (10.11)	4.40 (6.54)
Est (95% CI)	Referent	−0.82 (−1.14 to −0.49)	−1.33 (−1.61 to −1.05)	−1.61 (−1.89 to −1.33)	−1.73 (−2.01 to −1.44)	Referent	−1.38 (−1.69 to −1.07)
aEst^§^ (95% CI)	Referent	−0.84 (−1.17 to −0.52)	−1.36 (−1.65 to −1.08)	−1.66 (−1.94 to −1.38)	−1.77 (−2.05 to −1.49)	Referent	−1.44 (−1.75 to −1.12)
**Illness during past 2 weeks**							
Gastrointestinal	1,128 (6.0)	390 (4.9)	508 (4.8)	331 (4.6)	601 (4.1)	733 (6.1)	2,225 (4.7)
PR (95% CI)	Referent	0.82 (0.69 to 0.97)	0.81 (0.71 to 0.92)	0.77 (0.65 to 0.90)	0.68 (0.59 to 0.77)	Referent	0.76 (0.68 to 0.84)
aPR^§^ (95% CI)	Referent	0.82 (0.69 to 0.98)	0.81 (0.72 to 0.92)	0.78 (0.66 to 0.91)	0.69 (0.61 to 0.79)	Referent	0.77 (0.69 to 0.86)
Respiratory	2,736 (13.7)	1,045 (12.4)	1,316 (12.8)	919 (11.5)	1,892 (12.7)	1,687 (13.5)	6,221 (12.7)
PR (95% CI)	Referent	0.90 (0.82 to 0.98)	0.93 (0.86 to 1.01)	0.84 (0.76 to 0.93)	0.93 (0.86 to 1.00)	Referent	0.94 (0.88 to 1.00)
aPR^§^ (95% CI)	Referent	0.91 (0.83 to 0.99)	0.94 (0.86 to 1.02)	0.85 (0.77 to 0.94)	0.94 (0.88 to 1.02)	Referent	0.95 (0.89 to 1.02)

**Abbreviations:** aEst = adjusted estimate (from linear regression); aPR = adjusted prevalence ratio; CI = confidence interval; Est = estimate (from linear regression); FPL = federal poverty level; PR = prevalence ratio; SD = standard deviation.

* FPL represents an indicator used to define the boundary for those eligible for federal aid; FPL is defined by the U.S. Department of Health and Human Services annually each January to adjust for inflation and is proportional to the size of the household. Because the poverty line data includes both income and number of household members, there were more missing values for poverty level; therefore, the numbers in the below FPL and at or above FPL groups do not sum to the number in all income groups.

^†^ Data are presented as the unweighted number of respondents (N) and weighted percentage (%), with the exception of the mean number of school days absent (weighted mean and standard deviation presented).

^§^ Adjusted for age and sex of child, as well as year of data collection.

^¶^ Estimated difference from reference.

** Among those missing ≥1 school day only.

Results were similar when comparing children living below the federal poverty level with those at or above it. Children living below the poverty level were significantly less likely to have missed school during the past year (65% versus 71%) and also significantly more likely to have had a gastrointestinal illness (6% versus 5%) or respiratory illness (14% versus 13%) in the preceding 2 weeks (Table 2). Specifically, children living below the poverty level were 9% (95% CI = 7%–11%) less likely to have missed a day of school during the last year but were 23% (95% CI = 14%–31%) more likely to have had a gastrointestinal illness during the 2 weeks preceding the survey. The difference in respiratory illness between children below versus those at or above the federal poverty level was similar to results by income category (5%) but was not statistically different.

Among children whose parents or guardian reported respiratory or gastrointestinal illness during the preceding 2 weeks, the percentage who missed any school during the last year increased with increasing income level. Among children who had gastrointestinal illness, 84.2% (family income <$35,000), 84.3% ($35,000–$49,999), 88.1% ($50,000–$74,999), 86.2% ($75,000–$99,999), and 89.4% (≥$100,000) missed school in the past year. Similarly, 83.1% of children living below the poverty level with gastrointestinal illness missed school, compared with 87.4% of those living at or above the poverty level. Among children in the household income brackets listed above who had a respiratory illness during the preceding 2 weeks, 78.7%, 80.3%, 79.2%, 82.8%, and 83.7%, respectively, missed school, and 78.3% of children living in households below the federal poverty level missed school compared with 81.5% of those living at or above the poverty level. Differences for both gastrointestinal and respiratory illnesses were significant in final model risk ratios when grouped by federal poverty level, but not for all individual income categories.

When analyzed by the number of days missed in the last year, children in the lowest income bracket (<$35,000) missed a mean of 0.5–0.8 more days compared with children in other income brackets (Table 2). Among only those children who missed ≥1 school day, the differences were larger (mean = 0.8–2.1 more days). Similarly, overall, children living below the federal poverty level missed an average of 0.7 more days of school per year than did children in higher income households; among only those who missed ≥1 day of school, the mean difference increased to 1.4 days.

## Discussion

Compared with children from higher income households, those from lower income households were more likely to have had a gastrointestinal or respiratory illness during the 2 weeks preceding the survey. Although children from lower income households were less likely to have missed any days of school during the last year, those who did miss school missed more days than did children from higher income households.

The combination of increased illness prevalence and absenteeism with decreasing income status highlights the need for accessible, affordable resources and interventions at home and school. Multiple barriers faced by children in low-income households could explain these findings, including lack of access to preventive health care (*2*). Although targeted social distancing, such as a requirement for absence from school might be an effective recommended course of action to protect public health (*3*,*4*), low-income parents might not have the opportunities (e.g., paid sick leave from work) to be able to implement this. These circumstances might affect both their children’s ability to stay home from school and health-seeking behaviors (*5*). In the long-term, longer periods of absenteeism could be associated with adverse educational outcomes (*6*).

The findings in this report are subject to at least two limitations. First, although NHIS collects health and school absence data generalizable to the civilian noninstitutionalized U.S. population, the reasons for school absence are not collected. Second, both health and school absence data are parentally reported, making them subject to recall bias, and the data are not consistent in their respective recall timelines (preceding 2 weeks versus preceding year). However, recall of self-reported illness and school absenteeism is likely to be more accurate for the recent past (*7*); thus, the association between reporting of recent illness and school absenteeism is likely to be strengthened. In addition, subgroup estimates for illnesses, though small in difference (one percentage point) had relative standard errors <30.

From a public health perspective, these findings highlight a need for resources for, and attention to, preventive measures to keep children in school. Beyond practices in the home, schools have opportunities to serve as settings for preventing transmission of communicable diseases. Some school-based programs promoting handwashing, and more generally hand hygiene, have been found to be effective in reducing gastrointestinal and respiratory illnesses and associated absenteeism (*8*). Research suggests that peer support and provision of soap can increase handwashing and reduce absenteeism related to both gastrointestinal and respiratory illnesses (*9*). However, further study of sustained, community-based encouragement of proper hand hygiene practices as effective, low-cost means of preventing such illnesses is needed. Ongoing health promotion activities in schools, in addition to the home, can increase awareness and understanding of handwashing with soap as an effective and affordable way to prevent transmission of infectious diseases. Increased public awareness of the importance of hand hygiene, as promoted by Global Handwashing Day (observed each year on October 15), is important to promoting public health and reducing the transmission of illness.

**Summary**

**What is already known about this topic?**

Gastrointestinal and respiratory infections are important illnesses that affect U.S. school-aged children. Schools can serve as primary settings of transmission.

**What is added by this report?**

During 2010–2016, parents of children from low-income households were more likely to report recent childhood gastrointestinal and respiratory illnesses than were higher income parents. Although parents of children from low-income households were less likely to report missing any school, when these children did miss school, they tended to miss more school days, on average.

**What are the implications for public health practice?**

Further studies to explore what factors underlie these associations with income are needed. In the meantime, public health partners could expand efforts to prevent gastrointestinal and respiratory illnesses in schools, especially low-cost measures such as promoting hand hygiene education.

## Footnotes

* The federal poverty level represents an indicator used to define the boundary for those eligible for federal aid. It is defined by the U.S. Department of Health and Human Services annually each January to adjust for inflation and is proportional to the size of the household (e.g., by 2018 guidelines, a two-person household with an income of $15,500 would be below the poverty level, but a single-person household with the same income would not).
